# Cottage by the Sea

**Published:** 1874-02

**Authors:** 


### COTTAGE BY THE SEA.

Childhood's days now pass before me,
Forms and scenes of long ago,
Like a dream they hover o'er me,
Calm and bright as evening's glow,
Days that knew no shade of sorrow;
There my young heart pure and free,
Joyful hailed each coming morrow,
In the cottage by the sea;
Joyful hailed each coming morrow,
In the cottage, the cottage by the sea.

Fancy sees the rose-trees twining
Round the old and rustic door,
And below, the white beach shining,
Where I gathered shells of yore.
Hears my mother's gentle warning,
As she took me on her knee;
And I feel again life's morning,
In the cottage by the sea.
And I feel again life's morning,
In the cottage, the cottage by the sea.

What though years have rolled above me,
Though 'mid fairer scenes I roam,
Yet I ne'er shall cease to love thee,
Childhood's dear and happy home!

And when life's long day is closing,
  Oh! how pleasant it would be,
On some faithful heart reposing
  In the cottage by the sea.
On some faithful heart reposing
  In the cottage, the cottage by the sea

